# Student engagement profiles in a mobile app: Links to self-regulated learning and performance

**DOI:** 10.1007/s11423-026-10586-2

**Published:** 2026-01-21

**Authors:** Jacqueline Wong, Mohammad Khalil, Vsevolod Suschevskiy, Martine Baars, Bjorn de Koning, Fred Paas

**Affiliations:** 1 Department of Education, Faculty of Social and Behavioral Sciences, Utrecht University, Utrecht, the Netherlands; 2 Centre for the Science of Learning & Technology (SLATE), Faculty of Psychology, University of Bergen, Bergen, Norway; 3 Northwestern University, Illinois, USA; 4 Behavioural Science Institute, Radboud University, Nijmegen, the Netherlands; 5 Department of Psychology, Education, and Child Studies, Erasmus School of Social and Behavioural Sciences, Erasmus University Rotterdam, Rotterdam, the Netherlands; 6 School of Education, University of New South Wales, Sydney, Australia

**Keywords:** Self-regulated learning (SRL), Retrieval practice, Student engagement, Mobile applications, Trace data, Latent profile analysis

## Abstract

Engaging in independent learning during self-study time is an essential part of learning in higher education. The ubiquity of mobile devices and their applications offer students a flexible, on-the-go learning experience. However, flexible learning environments require students to self-regulate their learning. The current study examined student engagement with a mobile study app intended for students to engage in retrieval practice with feedback as part of self-regulated learning (SRL). Three research questions were addressed: (1) What are the student engagement profiles that can be identified based on students’ activity with the study app? (2) Do the profiles differ in SRL and academic performance? and (3) What is the relationship between the identified student profiles, SRL, and academic performance? Through a learning analytics approach, results identified three distinct student profiles: active, disengaged, and utilitarian. Self-efficacy and time-management positively predicted exam grades, while self-evaluation showed an inverse effect. Active engagement positively influenced exam grades, underscoring individual differences in SRL.

## Introduction

With mobile devices such as smartphones becoming more affordable, the overall acceptance of adopting and integrating them into higher education to support learning is becoming increasingly attractive (Hartley & Andújar, 2022). There is a wide interest in incorporating mobile applications for higher education including learning management and reinforcement of learning (Goundar & Kumar, 2022). A prominent advantage of mobile device-mediated learning resides in its profound temporal and spatial flexibility in affording learners to study at any location at any time they want (El-Hussein & Cronje, 2010). The temporal and spatial flexibility fits well with the nature of independent learning during self-study time, where students in higher education are expected to engage in learning activities without instructors’ supervision above and beyond their scheduled in-class activities (Doumen et al., 2014). However, it is important to acknowledge that learning with mobile devices is not without challenges. While it offers flexibility and accessibility, it also presents potential drawbacks that can impact the learning process.

Potential drawbacks of learning with mobile devices include distractions from notifications, difficulties in maintaining focus in varied environments, limitations of small screens, and the risk of encouraging shallow rather than deep learning (Criollo-C et al., 2018). Additionally, the very flexibility that makes mobile-device mediated learning attractive can lead to inconsistent study habits without proper self-discipline. These factors highlight a further need to investigate self-regulated learning (SRL) in the context of mobile device-mediated education. The autonomy of independent learning and mobile device-mediated learning demands SRL, a process in which students direct multiple aspects of their learning process, including motivation, cognition, metacognition, and behavior, toward personal learning goals (Zimmerman & Moylan, 2009).

Despite the critical role SRL plays in academic success across all educational levels (Broadbent & Poon, 2015; Richardson et al., 2012), research shows that students do not necessarily self-regulate their learning effectively, and their motivation can have an impact on the extent to which they use SRL strategies (Lee et al., 2020). Several studies have concluded that self-regulating one’s learning during independent learning is difficult and challenging since many students are poor at monitoring their learning and lack adequate knowledge and skills to use effective learning strategies (Bjork et al., 2013; Cervin-Ellqvist et al., 2021). One effective learning strategy with strong empirical support for its effectiveness is retrieval practice, also known as practice testing (for a review, see Carpenter, 2023). However, studies showed that while students list retrieval practice as a strategy that they often use, for example through quizzes and flashcards, they tend to employ it for assessing rather than improving their knowledge (Hartwig & Dunlosky, 2012). Consequently, this leads to the underutilization of retrieval practice, functioning merely as a one-time knowledge check after studying.

An approach to increase students’ use of retrieval practice is to provide direct and easy access to resources for retrieval, such as a study tool that students can use to take low-stakes quizzes at multiple times (Carpenter, 2023). Kang et al. (2023) highlighted the potential of technology in promoting retrieval practice as a learning strategy. In the study, undergraduates who attended a financial workshop were randomly assigned to one of the three conditions where a mobile app sent notifications for (a) spaced practice (i.e., students had one week to complete two quizzes), (b) massed practice (i.e., students had 24 h to complete two quizzes, or (c) a control condition where students were not given the two quizzes. The study showed that students in the spaced practice outperformed students in the massed practice and control condition, but there were no significant differences between the massed practice and control condition. Given that log data were not examined, it remained unknown to what extent students engaged with the quizzes within the time stipulated and whether students could take the quizzes multiple times. Students with poor motivation and SRL may miss learning opportunities when their engagement with the quizzes delivered by mobile apps is suboptimal, low, or non-existent (Nichter, 2021). Despite the wealth of research on retrieval practice, very few studies have examined how students engage with retrieval practice on their own when provided with a (mobile) application designed for this purpose.

The aim of the current study is twofold: 1) to identify student engagement profiles using the log data from a mobile app, and 2) to examine the relationship between the identified student engagement profiles, SRL, and learning performance. By employing a learning analytics approach to examine log data from a mobile study app using latent profile analysis, the results will offer insights into how different groups of students engage with the study app, paving the way for more targeted interventions to support less optimal forms of engagement. Furthermore, the use of multiple sources of data (i.e., log data, self-report, and exam grades) in this study helps to deepen our understanding of the relationship between student characteristics (i.e., SRL), academic performance, and student engagement in the context of mobile device-mediated learning.

### Facilitating self-regulated learning in mobile contexts

While various models of SRL have been proposed in research to theorize the strategies and processes that students engage in when self-regulating their learning, one of the most widely cited SRL models is three-phase cyclical model by Zimmerman and Moylan (2009; for review, see Panadero, 2017). The three-phase cyclical model consists of three main SRL phases: the forethought phase involves processes of task analysis and goal setting, the performance phase involves the use of effective learning strategies and monitoring of one’s progress, and the self-reflection phase involves processes of self-evaluation. In general, self-regulated learners can be characterized by three features: (a) use of SRL strategies, (b) adaptation of subsequent SRL activities based on feedback, and (c) interdependence with motivational processes (Zimmerman, 1990). Both will (i.e., motivation) and skill (i.e., cognitive) components of learning are integral to SRL (McCombs & Marzano, 1990).

Mobile applications can potentially be used to facilitate SRL, particularly the skill component, by providing students with extended opportunities to seek out learning, for example by using retrieval practice, since mobile devices (e.g., smartphones, tablets, e-readers) are almost always within one’s reach. Retrieval practice was ranked as a learning strategy with high utility by Dunlosky et al. (2013) based on its positive effects across different testing formats (e.g., recall, multiple-choice), learning tasks (e.g., word pairs), outcome measures, and age of students (for review, see Adesope et al., 2017). Despite the benefits of retrieval practice, research showed that students tend to rely on less effective strategies, such as rereading (Hartwig & Dunlosky, 2012). To increase the utilization of retrieval practice by students, leveraging mobile technology is a promising approach to encourage students to easily implement and use it as part of their learning repertoire.

Mobile devices have greatly influenced the way people communicate and learn. Historically, mobile learning became more popular in the early years of the twenty-first century. Usually referred to as m-learning, this domain encompasses learning experiences that are both time- and location agnostic, facilitated by using mobile computing devices to access educational content without the constraints of physical locations (Caudill, 2007; Wu et al., 2012). While the ubiquity of m-learning may offer learners the opportunity to take charge of their own learning processes, setting goals, monitoring progress, and making decisions about how to study, which are attributes of SRL, Sha et al. (2012) argued that SRL is a precursor as well as a desired outcome of m-learning. A systematic review by Palalas and Wark (2020) supports this argument when examining the relationship between m-learning and SRL. The reviewed studies suggest a complex and dynamic relationship between m-learning and SRL, whereby both factors work together to enhance learning. On the one hand, the affordances of mobile devices may enhance SRL for learners, pointing to m-learning’s potential to empower learners to adapt their learning strategies. On the other hand, SRL may enhance m-learning by optimizing the use of mobile devices for learning.

While mobile device-mediated contexts are well placed for SRL, digital learning platforms, environments, and/or applications need to be carefully designed to promote student engagement and optimize SRL. Studies in open online education suggest that students seldom use SRL support even when provided with one, as such, SRL interventions generally suffer from low compliance (Wong et al., 2021). Therefore, to understand the impact of SRL support, it is critical to examine how different students engage with it. The existing literature features a variety of mobile apps designed to support SRL (Baars & Viberg, 2022). For example, in Baars et al. (2022a, 2022b), a mobile app was developed to support SRL through cognitive and metacognitive prompts. Despite a large number of students who registered for a user account, only about a quarter of them created study sessions using the app. The low uptake could be an indication that many students did not see a need to use the app for SRL. A follow-up study was conducted with undergraduate students and results showed that while students were positive about the app, they felt that they would only need to use it just before an exam or only if their grades would be insufficient (Baars et al., 2022a, 2022b). These studies set an example of the importance of examining the relationship between SRL and students’ engagement with study apps to understand better the impact of m-learning (or the lack thereof).

### Self-regulated learning and academic performance

The low compliance in SRL interventions also indicates that skill is necessary but not sufficient. Students need to be willing and motivated to self-regulate their learning (Zimmerman, 1990). Academic motivation has been shown to be predictive of SRL, and consequently, academic achievement (Manganelli et al., 2019; Metallidou & Vlachou, 2007; Richardson et al., 2012). Many models of motivation may be relevant for SRL, the current study focuses on two types of academic motivation: self-efficacy (beliefs about one’s academic capabilities) (Zimmerman, 2000) and achievement goal complexes (an integration of type of goals, namely mastery and performance, and reasons for goal pursuit, namely autonomous and controlled) (Liem & Senko, 2022).

The positive impact of self-efficacy on SRL and academic performance is well-established in research (Sitzmann & Ely, 2011). Highly self-efficacious learners are more likely to set challenging goals, use effective learning strategies and persist in a task. Successful learning experiences can enhance self-efficacy while negative experiences, such as attempting difficult tasks, can diminish self-efficacy (Pintrich, 1999). Self-efficacy promotes SRL behavior and plays a key role in understanding the likelihood of engaging in self-regulatory learning activities, such as using a study app for retrieval practice (Rachels & Rockinson-Szapkiw, 2018).

Achievement goal orientation and self-determination theory are both influential theories to explain motivation and academic success (Ciani et al., 2011; Lee et al., 2020). Sommet and Elliot (2017) connected these theories by proposing achievement goal complexes as a self-motivational framework to study what individuals want to achieve (i.e., goal orientations) and the reasons connected to the goals (i.e., autonomous or controlled reasons). The achievement goal complex framework integrates achievement goal theory and self-determination theory to examine combinations of goal orientations (i.e., mastery goals focus on gaining personal competence while performance goals focus on outperforming peers) and motives underlying the goals (i.e., autonomous reasons are regulated by intrinsic or identified motives while controlled reasons are regulated by introjected and external motives). The combination results in four achievement goal complexes: 1) autonomous mastery goal (AMG), 2) autonomous performance goal (APG), 3) controlled mastery goal (CMG), and 4) controlled performance goal (CPG). The four goal complexes aim to offer a more comprehensive account of academic motivation to understand the goals and reasons that influence one’s SRL (Tan et al., 2022).

Arguably, student’s academic motivation may influence their choice and effort in engaging with the study app. Also, the extent to which they engage and learn with the study app may influence the relationship between motivation and achievement. Therefore, self-motivational variables (i.e., self-efficacy and achievement goal complexes) are included to examine the relationship between SRL, academic performance, and student engagement.

### Employing learning analytics to examine student engagement profiles

Learning analytics is a field that is concerned with measuring, collecting, analyzing, and reporting of data to improve students’ learning experiences and to optimize learning and the environment in which it occurs (Long & Siemens, 2011). Students leave traces when they interact with online learning environments. Since trace data are generated when students apply their cognition while working on an online task, trace data can be considered observable indicators of cognition (Winne, 2022). For example, Winne et al. (2019) proposed the use of the nStudy software system to explore how trace data reflects SRL, highlighting the potential of digital footprints as observable and quantifiable indicators of students' cognitive processes. The nStudy software system includes digital highlighting and notetaking tools to support SRL and captures trace data in the form of students’ interactions with the support features as they engage in the learning activities to gain information for learning analytics. Our study extends this approach by employing learning analytics to analyze log data from a mobile study app designed to support retrieval practice and identifying distinct engagement profiles that reflect varying SRL strategies (e.g., how early one starts to engage with the study app) and their impact on academic performance. Our approach is grounded in studies showing that trace data (e.g., the number of logins, interactions with course materials) explain significant variance in academic achievement (Gašević et al., 2015; van Halema et al., 2020).

Studies employing learning analytics have shown that there is a strong relationship between self-reported SRL and students’ engagement patterns obtained from log data in the online learning environment. For example, Kizilcec et al. (2017) reported that students with higher SRL revisited course materials more than students with lower SRL. Research in SRL support also showed that supporting SRL increases student engagement with online course materials (e.g., Alemayehu & Chen, 2023). Such studies used a variable-centered approach that assumes students are similar in the way they engage with the course materials. However, individual differences exist in SRL (for review, see Wong et al., 2019). Therefore, van Alten et al. (2021) proposed investigating SRL using a person-centered approach that assumes heterogeneity in the population to identify patterns or profiles based on shared similar characteristics of a subgroup of students. A person-centered approach can be particularly useful in understanding whether there is a possibility for a subgroup of students who benefitted more than another subgroup when learning in the same online environment or when provided with the same study support tool.

Profiling students according to their interaction and engagement has been emerging in the research studies of learning analytics. For example, Kim et al. (2018) used learning analytics techniques, specifically k-medoid clustering on log data from 284 undergraduate students enrolled in an online statistics course, to identify SRL profiles as well as examine SRL learning behaviors. To carry out the clustering, the researchers looked at seven attributes from the online course: time spent on online lectures, online lecture access frequency, login interval regularity, online lecture access interval regularity, time spent on the Q&A board, Q&A board visit frequency, and the number of messages posted on the Q&A board. Using Principal component analysis (PCA), the clustering techniques resulted in three SRL-based groups following a good variance in the data: self-regulation group, partial self-regulation, and non-self-regulation. Translating each group’s behavior helped to advance the field’s “understanding of how students with different SRL profiles behave differently throughout the entire course” (p. 248). While this study provides a thorough examination of unknown but existing SRL patterns, it only focused on quantitative data analysis. Focusing solely on log data, this study did not consider whether students’ perceptions, such as motivation, differ across the identified profiles. Including student perceptions through qualitative data collection and analysis can draw a bigger picture of understanding complex interplay among perceptions of SRL and student behavior in digitalized environment.

### Current study

The current study was implemented in a nine-week undergraduate Law course at a major university in the Netherlands. Students in the course consisted of both part-time and full-time students. Attending on-campus lectures was optional for all students. Students in the full-time program had to attend nine mandatory on-campus tutorial meetings. A mobile app developed by a team consisting of a university lecturer, developer, and programmer was used in the course as an optional study tool.

The mobile study app was designed to enhance learning by facilitating SRL through the use of retrieval practice. To use the app in a course, the course instructor had to provide a set of multiple-choice questions (MCQs) along with the answers and explanations. Given that 60% of the final exam grades are based on students’ MCQ scores, the use of MCQs in the mobile study app provides students with ample practice to reinforce key information (e.g., recall of legal principles) through retrieval practice and helps to familiarize students with the MCQ type of exam questions. The use of MCQs in the mobile app is purposeful since MCQs are often used in courses with a high number of students, such as law, for practical reasons. Additionally, the format of MCQs offers students convenience, as students can engage with them easily using the mobile study app on the go.

**Fig. 1:**
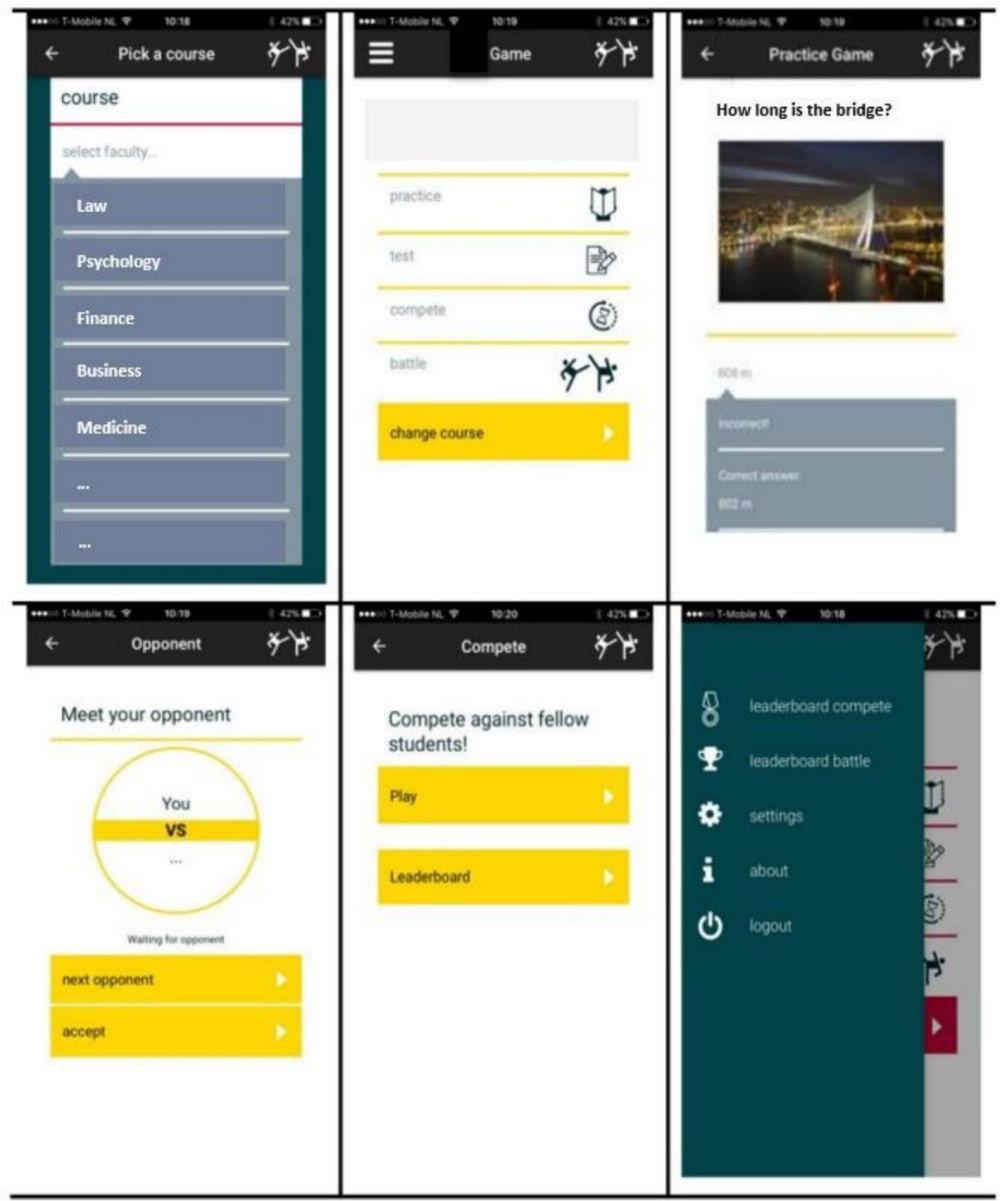
Snapshots of main features in the mobile study app

Figure 1 provides snapshots of the main features of the mobile app. There were four ways in which students could use the study app as a learning tool: practice, test, compete, and battle. When students selected practice, they could answer the MCQs and receive immediate feedback after each MCQ (i.e., whether the answer was correct or wrong along with a short explanation). In the test mode, students would answer a set of MCQs and receive feedback only at the end of the set of MCQs. For the compete mode, students would compete with other students by correctly answering as many MCQs as possible within a set time. A wrong answer would end the competition, and the score would be shown on a leaderboard. For the battle mode, students could select an opponent (e.g., a peer or an instructor) in the online environment to a challenge of correctly answering the greatest number of MCQs in the shortest time.

Given that using the study app was optional, we were interested in examining how students engage with the app and whether students could be clustered into subgroups based on their engagement level. The main research question was formulated as “What are the student engagement profiles that can be identified from students’ activity in the mobile study app?” (RQ1). Due to the exploratory nature of the study, we did not have any specific hypotheses on the number of student engagement profiles. However, given the number of profiles found in previous studies on student engagement, we expected to find between two to four types of student engagement profiles (Khalil & Ebner, 2017; Tseng et al., 2016).

Additionally, two research questions were formulated to investigate the interplay between perceived SRL, student engagement behavior, and academic performance: “To what extent do the identified profiles differ in their perceived SRL and academic performance?” (RQ2) and “What is the relationship between the identified student engagement profiles, SRL, and academic performance?” (RQ3). Previous research suggests that students with higher SRL are more engaged in their learning and achieve higher exam grades than those with lower SRL (Manganelli et al., 2019; Metallidou & Vlachou, 2007; Zimmerman, 2000). Therefore, we hypothesized that the perceived SRL would be higher for profiles with higher engagement than profiles with lower engagement. Similarly, profiles with higher engagement will have higher exam grades than profiles with lower engagement. We also expected to find a positive linear relationship between perceived SRL, student engagement, and academic performance.

## Method

### Participants

The participants were 310 undergraduates taking a course in Law from a university in the Netherlands. Of these, 303 (males, *n* = 95; females, *n* = 208) completed the survey. The mean age of the sample was 21.1 years old (*SD* = 5.2); males (Age_mean_ = 21.3, Age_sd_ = 4.4), females (Age_mean_ = 21.1, Age_sd_ = 5.5). Participating in the study was voluntary and participants were not compensated for their participation. The study followed the university’s ethical committee guidelines.

### Measurement

#### Self-regulated learning

Five sets of items were used to measure SRL, two for motivation, namely self-efficacy (8 items) and goal complexes (24 items), one for metacognitive and effort regulation (8 items), one for time-management (8 items), and one for self-evaluation (6 items). All items were measured on a Likert scale ranging from 1 (not at all true) to 7 (very true). The subscales were put together from different sources as described below with example items provided for each subscale.

The 24 goal complex items were adapted from Sommet and Elliot (2017) and consisted of four subscales: four items in autonomous-mastery (AMG; “My goal is to *learn as much as possible* because I find this a highly stimulating and challenging goal.”), four items in autonomous-performance (APG; “My goal is to *perform better than the other students* because I find this a highly stimulating and challenging goal.”), eight items in controlled- mastery (CMG; “My goal is to *learn as much as possible* because I would feel bad, guilty, or anxious if I didn’t do it.”), and eight items in controlled-performance (CPG; “My goal is to perform better than the other students because I would feel bad, guilty, or anxious if I didn’t do it.”). The Cronbach’s alpha for AMG, APG, CMG, CPG are .82, .84, .86, .90 respectively in the current study. Self-efficacy was adapted from the Motivated Strategies for Learning Questionnaire (MSLQ; Pintrich et al., 1991) with a Cronbach’s α of 0.89. An example item is “I believe I will receive an excellent grade in this class.”.

The metacognitive and effort regulation scale was modified from MSLQ and adapted from Dunn et al. (2012). Five items measure general strategies for learning (GSL; “When studying for this course, I make up questions to help me focus my reading.”) and three items were used to measure clarification strategies for learning (CSL; “When I become confused about something I am reading for this course, I go back and try to figure it out.”). The Cronbach’s alpha for general strategies for learning and clarification strategies for learning were .61 and .50 respectively.

The time-management scale was also adapted from the MSLQ (Pintrich et al., 1991) with a Cronbach’s α of .76. An example item is “I usually study in a place where I can concentrate on my coursework”. The self-evaluation scale was adapted from the metacognitive awareness inventory in Schraw and Dennison (1994). An example item is “I ask myself if there was an easier way to do things after I finish studying a topic in this course.”. One item (“I know how well I have learned once I finish studying a topic in this course”) was removed to improve the internal reliability from α =.68 to α =.70.

#### Academic performance

Academic performance was measured using exam grades provided by the course instructor. The final exam consisted of multiple-choice questions with a weightage of 60% and open-ended questions with a weightage of 40%. The exam grades were calculated based on students’ weighted scores across the two exam components. The maximum points that could be obtained was 100.

#### Student engagement indicators

To examine the different ways in which students engaged with the mobile app, we identified ten indicators from log data as proxies for student engagement (Kim et al., 2018). Table 1 provides an overview of the ten indicators.

**Table 1:** Description of the ten student engagement indicators operationalized from the mobile study app with the overall range and median

Indicator	Operationalization
Attempts	The number of MCQs students attempted to answer, regardless of the result. The number of attempts varied from 1 to 4090, with a median of 208
Successful attempts rate	The percentage of attempts correctly answered. Successful attempts range between 16.7% and 100% with a median of 71.5%
Unique days	The number of unique days that students used the app. The unique days ranged from 1 to a maximum of 20 days, with a median of 3
Consecutive day	The maximum number of consecutive days that students used the app as an indication of continuous learning. This metric runs from 1 to 7 with a median of 2
Early start	The difference between the first date when students started to use the app and the date of the exam. The minimum number of days before the exam is −5 (i.e., five days after the exam) and the maximum is 35 days, with a median of 11 (i.e., days to the exam)
Late finish	The difference in days between the last date students used the mobile app and the date of the exam. The minimum number of days is −5 (i.e., five days after the exam) and the maximum is 32 days, with a median of one day before the exam
Maximum time	The maximum time spent on a single question. The maximum time spent on a single question ranged from 8 to 119 s, with a median of 95 s
Minimum time	The minimum time spent on a single question. The minimum time spent on a single question ranged from less than 1 to 26 s, with a median of 1
Mean time	The mean time spent on a single question. The mean time spent on a single question across all participants ranged from 2 to 33 s, with a median of 9.5 s
Use of compete and battle modes	The percentage of attempts in using the compete and battle modes over all attempts. Most students did not use these modes at all resulting in a median of 0%, but the third Quartile is only 25%, with a maximum of 95%

### Study procedure

Announcements to invite students to participate in the study were made by the course coordinator and tutors in the first two weeks of the course. Students were provided with a survey link to access the questionnaire hosted on a survey platform, Qualtrics (www.qualtrics.com). Students were initially asked for consent to collect data, including survey responses, mobile app usage, and course grades. They subsequently completed a questionnaire on demographics, motivation, and SRL. The mobile app was available to students throughout the course, and its use for independent learning during self-study time, while encouraged, was optional. Data analysis involved collecting mobile app usage and course grades at the end of the course.

### Analytical procedure

For the Latent Profile Analysis (LPA), we used the tidyLPA package to conduct the analysis in R (Rosenberg et al., 2019). The ten student engagement indicators were standardized using proportion of maximum scoring (POMS) (Little, 2013), as such, each scale ranges from 0 to 1, indicating the minimum and maximum possible values. POMS maintains the distance between observations while keeping the data structure intact to enable easier comparison between different indicators. The models were then evaluated by a number of fit criteria and statistics for which the best-fitting model is indicated by the lowest values in Akaike's Information Criterion (AIC), Approximate Weight of Evidence (AWE), Bayesian Information Criterion (BIC), Classification Likelihood Criterion (CLC), and Kullback Information Criterion (KIC) (for a detailed description on determining the number of clusters based on the fit criteria, see Akogul & Erisoglu, 2017). To ascertain differences in student engagement behaviors between the profiles, non-parametric Kruskal–Wallis tests were employed with the student engagement profile membership as the independent variable and the ten indicators of student engagement as dependent variables.

To examine the relationship between student motivation, SRL, exam grades, and student engagement profiles, we first conducted a confirmatory factor analysis for the latent variables measuring perceived SRL (i.e., AMG, APG, CMG, CPG, self-efficacy, metacognitive and effort regulation, time-management, and self-evaluation). Based on the factor scores derived from the confirmatory factor analysis, we examined differences in SRL, and exam grades among the identified student engagement profiles using separate non-parametric Kruskal–Wallis tests.

Given that several latent variables were highly correlated, and the fit indices were outside the acceptable range, we stepwise excluded model parameters, taking a data driven approach. We used an MLR estimator (maximum likelihood estimation) with robust standard errors and FIML missing data estimator as a more efficient and accurate alternative to list-wise deletion (Enders & Bandalos, 2001). The final confirmatory factor analysis model obtained a CFI of 0.747, which shows a less than acceptable fit. The Chi-square, RMSEA and SRMR values (*p* < 0.001, *RMSEA* = 0.074, *SRMR* = 0.077) suggest a marginally acceptable model fit (for more information on fit indices, see Kline, 2005 and Hooper et al., 2008). The final confirmatory factor analysis model included only 3 latent variables that were correlated with final grades: time management (7 items), self-evaluation (5 items), and self-efficacy (8 items).

In the final step, the identified profiles and latent variables that were highly correlated with final grades were fitted in a multiple linear regression model. A combination of the top-down and step-up strategy was used to build the final model, and the model was diagnosed with the “performance” package in R (Lüdecke et al., 2021). The full model was initially built with all latent variables. Then, insignificant predictors were removed in a stepwise process before subsequently adding interaction effects. The results from the bootstrapped regression analysis did not alter the interpretation of any key model parameters compared to the original ordinary least squares estimates. The detailed output of the bootstrapped model is included as a robustness check. The study materials, anonymized data, and code used for the analysis can be accessed via https://osf.io/qe7p5.

## Results

### Student engagement profiles

**Fig. 2:**
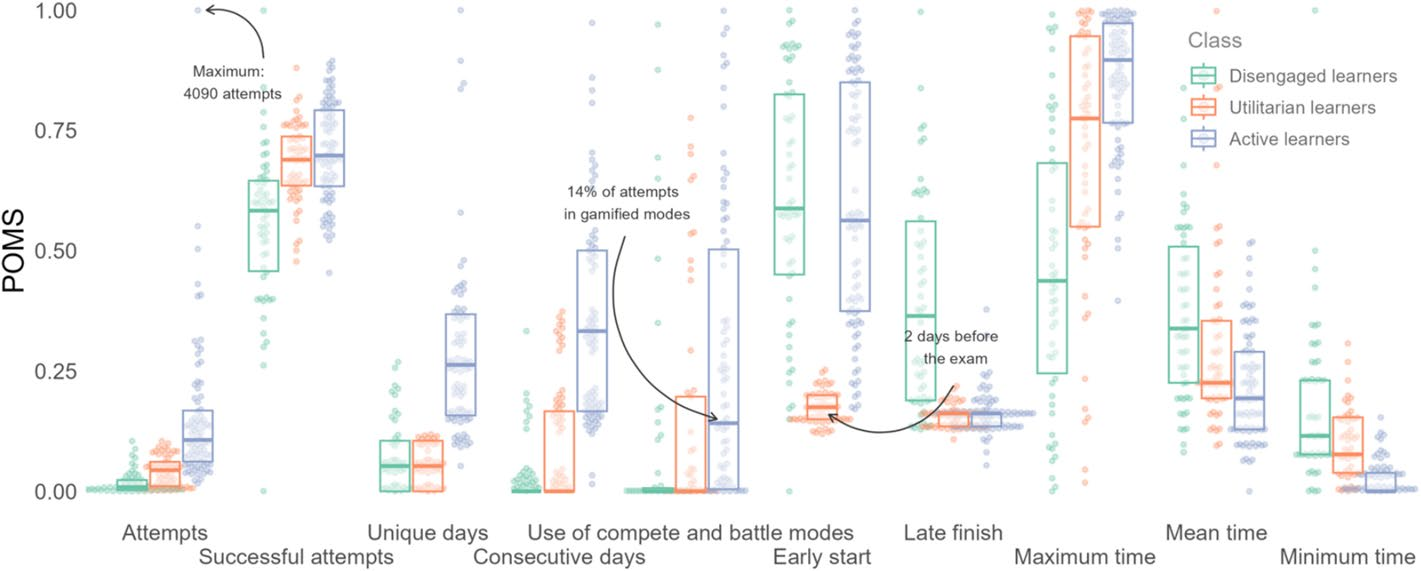
Comparison of the ten student engagement indicators across the three profiles

Results of the latent profile analysis on the ten student engagement indicators revealed that Model 2 (i.e., varying variances and covariances fixed to zero) with three profiles had the lowest value for AIC (−2549), AWE (−1906), BIC (−2367), and KIC (−2490) while Model 6 (i.e., varying variances and varying covariances) with two profiles had the lowest value for CLC (−2752). Therefore, Model 2 with three profiles appeared to be the best-fitting model. Figure 2 shows the differences in the ten student engagement indicators across the three profiles. Median value of the ten student engagement indicators across the three profiles are shown in Table 2.

**Table 2:** Median value of the ten student engagement indicators across the three profiles

Student engagement Indicators	Cluster 1: Disengaged *n* = 54	Cluster 2: Utilitarian *n* = 57	Cluster 3: Active *n* = 82
Attempts	36.5	181.0	438.0
Successful attempts	65.2%	74.1%	74.8%
Unique days	2.0	2.0	6.0
Consecutive days	1.0	1.0	3.0
Early start	18.5	2.0	17.5
Late finish	8.5	1.0	1.0
Maximum time	56.5	94.0	107.5
Minimum time	3.0	2.0	< 1
Mean time	12.5	9.0	8.0
Use of compete and battle modes	0%	0%	14%

The first profile (*n* = 54) is labeled as ‘disengaged’. Students in this profile attempted the least number of questions and had the least successful attempts. They started using the app early in the course (i.e., 18.5 days to the exam) but also stopped using it well before the exam (i.e., 8.5 days to the exam). While they spent more time on average answering each question, they did not spend as much time working on a question. Specifically, they spent 12.5 s on average answering questions compared to the active users who spent 8 s on average. However, the maximum time spent on a question for the disengaged users was 56.5 s compared to the maximum time of 107.5 s spent on a question by the active users. The engagement pattern suggests that they were not actively engaging with the study app and were unlikely to be using it to prepare for their exams.

The second profile (*n* = 57) is labeled as ‘utilitarian’. Students in this profile attempted a moderate number of questions. Unlike the active and disengaged users, they only started using the app close to the exam date (i.e., 2 days to the exam), suggesting that they were using the app as a tool to practice for their exams. The time engagement pattern was similar to the active users, they spent on average less than 10 s on a question, but the maximum time suggests that they also spent some time over questions that were more challenging for them.

The third profile (*n* = 82) is labeled as ‘active’. Students in this profile attempted the greatest number of questions.. They also spent the most number of unique days in the app and used it for three consecutive days. They started using the app early from 17.5 days to the exam and kept using it till the day before the exam. When observing the time they spent on answering the questions, the maximum time was the highest and minimum time was the lowest, suggesting that they were quick to answer the questions but also put in time to deliberate over questions that were more challenging for them. On average, they spent less than 10 s on a question. It is also interesting to note that they were the only group that spent 14% of their attempts on the compete and battle modes in the study app.

Apart from the three student profiles that were identified, students who completed the survey but did not use the mobile app at all were included in further analyses. This group of students is regarded as the fourth profile and labeled as non-users (*n* = 117).

### Differences in SRL and academic performance

Table 3 shows the median values for the perceived SRL and exam grades based on the available data across the three identified student engagement profiles (disengaged, utilitarian, active) and non-users, and the *p*-values of corresponding non-parametric Kruskal–Wallis test. Median values were chosen as an alternative to the means to reflect the real structure of non-normally distributed data (e.g., active users have a higher median value on time-management than other profiles).

**Table 3:** Median value of perceived srl and exam grades across three identified student engagement profiles and non-users

Factor	N^1^	Non-users	Disengaged	Utilitarian	Active	*p*-value^2^
	310	*n* = 117	*n* = 54	*n* = 57	*n* = 82	
Autonomous mastery goal (AMG)	251	0.25 (−0.58, 0.53)	0.25 (−0.58, 0.53)	−0.03 (−0.58, 0.53)	0.25 (−0.44, 0.81)	0.2
Autonomous performance goal (APG)	251	−0.09 (−0.99, 0.63)	0.27 (−0.63, 0.99)	0.09 (−0.45, 0.81)	0.45 (−0.72, 0.99)	0.045
Controlled mastery goal (CMG)	251	0.00 (−0.43 0.74)	0.10 (−0.64, 0.84)	−0.16 (−0.85, 0.63)	−0.11 (−0.95, 0.79)	0.4
Controlled performance goal (CPG)	251	−0.27 (−0.76, 0.72)	0.42 (−0.76, 1.21)	−0.17 (−0.76, 0.72)	−0.27 (−0.81, 0.72)	0.4
General Strategies for Learning (GSL)	223	0.09 (−0.64, 0.57)	−0.15 (−0.88, 0.57)	0.33 (−0.39, 0.81)	0.33 (−0.39, 1.06)	0.056
Clarification Strategies for Learning CSL)	223	−0.01 (−0.76, 0.75)	−0.01 (−0.76, 0.75)	−0.01 (−0.57, 0.75)	−0.01 (−0.76, 1.12)	0.6
Self-efficacy	303	0.00 (−0.32, 0.31)	0.02 (−0.46, 0.48)	0.03 (−0.44, 0.36)	0.02 (−0.42, 0.42)	> 0.9
Self-evaluation	303	0.01 (−0.24, 0.24)	−0.02 (−0.35, 0.20)	−0.04 (−0.29, 0.33)	0.12 (−0.10, 0.38)	0.2
Time-management	303	0.00 (−0.19, 0.19)	−0.03 (−0.20, 0.08)	0.00 (−0.27, 0.19)	0.05 (−0.12, 0.28)	0.077
Final grade	279	56 (43, 68)	59 (48, 69)	65 (57, 73)	68 (57, 75)	< 0.001
^1^The sample size differs per variable due to missing data ^2^Kruskal-Wallis rank sum test						

Results of the Kruskal–Wallis tests showed significant differences between groups for autonomous performance goal (APG, χ^2^ = 8.02, *df* = 3, *p*_adj_ = 0.045) and exam grades (final grade, χ2 = 22.997, *df* = 3, *p*_adj_ < 0.001). Pairwise comparisons with Bonferroni correction were carried out for APG and final grades to examine the differences between profiles. Bonferroni results show that pairwise comparisons were significant only for the exam grades. None of the pairwise tests for APG were significant. Consequently, we excluded correlational assumptions for APG.

For exam grades, post hoc pairwise comparisons showed that active users obtained higher exam grades than non-users (*p*_adj_ < 0.001). Similarly, utilitarian also achieved a higher grade than non-users (*p*_adj_ = 0.009). There were no significant differences in exam grades between disengaged and non-users (*p*_adj_ = 1.00), utilitarian (*p*_adj_ =.737), and active users (*p*_adj_ =.092), nor between utilitarian and active users (*p*_adj_ = 1.00).

### Relationship between SRL, student engagement profiles, and exam grades

Table 4 shows the results of the multiple linear regression analysis. After removing cases with missing data using listwise deletion, the final analysis consisted of 266 observations. The active and utilitarian profiles have a significant positive correlation with final grades. Additionally, self-efficacy and time management are significantly and positively associated with the final grade. While self-evaluation is also a significant predictor of the final grade, the relationship is inversed.

**Table 4:** Results of the final multiple linear regression model

	Dependent variable: final grade
Disengaged	2.926 (2.373)
**Utilitarian**	8.989^***^ (2.227)
**Active**	10.221^***^ (2.045)
**Self-evaluation**	−12.204^***^ (3.109)
**Self-efficacy**	10.692^***^ (2.174)
**Time-management**	14.281^**^ (4.523)
Disengaged: Self-evaluation	−2.831 (5.668)
Utilitarian: Self−evaluation	3.649 (4.635)
Active: Self-evaluation	0.554 (4.646)
Disengaged: Self-efficacy	−3.660 (3.961)
Utilitarian: Self-efficacy	−5.689 (3.832)
**Active: Self-efficacy**	−14.022^***^ (3.669)
Disengaged: Time-management	−2.071 (7.642)
Utilitarian: Time-management	−9.116 (7.700)
**Active: Time-management**	21.519^**^ (7.985)
**Constant**	54.770^***^ (1.300)
Observations	266
Adjusted R2	0.319
Residual Std. Error	12.80 (*df* = 250)
F Statistic	9.27*** (*df* = 15; 250)
Note: *p < 0.1; **p < 0.05; ***p < 0.01	

Additionally, for the active profile, we observe an interaction effect with self-efficacy and time management on exam grade. Specifically, each increase in the time management score corresponds to an approximate 21-point advantage for students with the highest scores compared to those with the lowest scores. In contrast, interaction with self-efficacy displays a negative coefficient, indicating that students who use the app more frequently and score higher on the self-efficacy factor tend to have lower final grades on average than those with lower self-efficacy levels.

## Discussion

The current study took a person-centered approach to empirically investigate a mobile study app where students can engage in retrieval practice in four different ways: practice, test, compete, and battle. The main aim of the study was to examine whether students differ in the way they engage with the app by identifying student engagement profiles based on the indicators derived from the app’s log data (RQ1). Furthermore, to understand the interplay of SRL, student engagement with the study app, and academic performance, we investigated whether the student engagement profiles differ in their SRL and academic performance (RQ2) and the relationship among the three components (student engagement profiles, SRL, and academic performance) (RQ3).

### Student engagement profiles

Latent profile analysis based on ten student engagement indicators revealed three distinct profiles: *disengaged, utilitarian, and active.* The profiles are characterized by vastly different levels of engagement. Compared to disengaged users, utilitarians and active users had high attempt rate. Yet, the time point at which they engaged with the app was different. Utilitarians used the app closer to the exam date, potentially as a tool to check their understanding before exams (Bjork et al., 2013). The active users used the app over the duration of the course and were working with the app over more consecutive days. However, it is not clear if they also used it to check their understanding or as a way to employ retrieval practice during independent learning. Students in all three profiles spent similar amounts of time per question and were fast to answer a question (i.e., under 3 s). However, the maximum time on a question is much higher for the active users than the disengaged users. This suggests that active users might be more persistent in working on a question compared to disengaged users. Additionally, active users used the compete and battle modes more often than disengaged users and utilitarians, potentially indicating that they took one step further in their learning to challenge themselves with others. Future studies could include interviews to gain deeper understanding of whether and how students use such apps to improve their learning. Another direction is to include other log indicators that could provide insights into the strategies used, for example, interleaving of questions related to different topics (Kang et al., 2023).

Of the 303 students who completed the survey, one-third of them (*n* = 117) did not use the app at all. One possible reason is that students struggled with using the app. Cai et al. (2017) pointed out that usability issues can drive poor engagement. Regardless of usability issues, not using the study app means that students cannot reap the potential benefits the study app can bring. Therefore, more should be done to inform students about the utility of the app and to encourage them to use the study app for retrieval practice, for example, through explicit instruction and training (Carpenter, 2023). To better understand students’ reasons for not engaging with the mobile app, future studies could adopt a mixed-methods study approach to gather qualitative insights into the barriers to engagement.

### Student engagement profiles, SRL, and academic performance

The higher exam grades of the active and utilitarian users compared to non-users highlight the positive effect of using the mobile app on academic performance. Moreover, the lack of significant differences between the utilitarian, disengaged, and active users suggest that the app could benefit users irrespective of how they use it (e.g., right before the exam or over the duration of the course).

Aligned with prior studies (Sitzmann & Ely, 2011), time-management and self-efficacy were shown to have a positive effect on exam grades. In contrast, self-evaluation was negatively associated with exam grades. One possible explanation could be the self-evaluation bias (Narciss et al., 2011). Research shows that students are generally inaccurate at assessing their own performance (Kostons et al., 2010). Therefore, some students may have overestimated the extent to which they had mastered the content, believing they were well-prepared for the exams when they were not, which could have led to lower exam performance. Another explanation is that students with high levels of self-evaluation may be overly critical when assessing their own learning, leading to greater exam anxiety and, in turn, lower performance. Future research could explore whether a study app, such as the one used in the current study, can support and enhance students’ self-assessment accuracy.

Given that the mobile app is positioned as an optional study tool, it is not surprising that time-management plays a critical role. Among the active users, those with self-reported time-management achieved higher exam grades than those with low self-reported time-management. This suggests that students who are better at managing their time could have used the app more consistently and strategically to enhance their learning. One suggestion to enhance learning in the course is to integrate using the app as a study activity in the course. This approach could benefit students who are poor at managing their time. We must also consider the nature of mobile interactions which may not facilitate deep comprehension due to difficulties in maintaining focus and limitations of small screens (Ilić Semiz et al., 2024). The on-the-go nature of app usage, particularly evident for the utilitarian profile, might not always translate to long-term learning. Future research could look into the extent of knowledge gained through mobile apps compared to traditional study methods.

Another noteworthy finding from our study revealed an unexpected relationship between self-efficacy and final grades for the engagement profiles. Contrary to our expectations, self-efficacy demonstrated a significant negative correlation with final grades for the active learners. This unexpected result offers valuable insights into the complex interplay of motivation, app use, and academic achievement. One plausible interpretation of the negative correlation between self-efficacy and final grades is the presence of *overconfidence* among students who reported higher levels of self-efficacy. Perhaps these individuals who might exhibit a strong belief in their capabilities have been more inclined to underestimate the effort required for academic achievement (i.e., final grade). As a result, it may suggest that students took unnecessary risks in their study strategies. This overconfidence, according to our interpretation, may have led to lower final grades. Our finding aligns with Davis’ (2009) study, where self-efficacy negatively moderated the relationship between final exam scores and overall GPA. Given practical constraints, such as tight course schedule and limited access to students for completing surveys at multiple time points, self-efficacy was examined only at the start of the course in our study, as such, it is not clear if using the app had an impact on students’ self-efficacy. The app features (e.g., feedback and gamification), while not examined here, may have reinforced motivation and SRL, future studies could compare the reinforcing effects of such features. Furthermore, given the potential reciprocal influence of motivation and SRL on students’ engagement with the mobile study app, future studies could consider measuring motivation and SRL, such as self-efficacy and time-management over time to gain deeper insights into the impact of app use on self-efficacy.

### Limitations

There are several limitations in the study. The first is the limited selection of student engagement indicators. The ten student engagement indicators were selected by considering potential values (e.g., how early one starts and how late one stops using the app) indicative of the quality of engagement. We acknowledge that there can be other student engagement indicators that may provide valuable insights. Future research could consider student engagement indicators by examining the predictive value of each indicator on academic performance. Building on Winne et al.’s (2019) work on nStudy, future research could also explore the implementation of other app features (e.g., tagging questions for revision) or examine specific log indicators to further align with SRL processes, such as time spent on feedback explanations in the mobile app. A second limitation is the way the study app was positioned as optional in the course. Therefore, it is possible that students who needed support to engage in SRL activities to benefit their learning, did not choose to do so (Wong et al., 2021). Vice versa, students using the app might already be motivated or have better SRL skills. Furthermore, the study was only conducted in one course at one university, limiting the findings’ applicability to other contexts. Future research could investigate whether making app use mandatory for all students would yield similar user profiles, and whether comparable differences in SRL and academic performance can be found in other courses. A third limitation is that the mobile study app provides only MCQs. The ease in scoring MCQs makes MCQs an easy-to-implement question type. However, while MCQs are often used in assessments, arguments exist on whether they can assess higher-order cognition (Liu et al., 2024). With the emergence of generative AI technologies and powerful natural language processing capabilities, future research could investigate using large language models to enable retrieval practice on open-ended questions in such mobile apps.

## Conclusion

The current study employed a learning analytics approach to examine how students engaged with a mobile app that offers them the opportunity to employ retrieval practice as a study strategy. By analyzing log data, the study adds insights into the extent to which students engaged with the app – insights that otherwise would be difficult to obtain, such as in Kang et al.’s (2023) study. While student engagement was operationalized by only ten indicators (e.g., number of attempts), our findings advance our understanding of individual differences in students’ engagement with the study app and the impact on exam grades. Taken together, the results suggest that using the study app can benefit learning, but more needs to be done to improve the implementation and awareness of the utility of the mobile app. Additionally, identifying student engagement profiles provides a first step towards developing tailored interventions to support students with different student engagement profiles, for example, nudging disengaged users through app notifications to increase engagement with the mobile app. The study contributes to the use of learning analytics in understanding the application of SRL theories in the context of mobile device-mediated learning.

## Data Availability

The data that support the findings of this study are openly available in OSF at 10.17605/OSF.IO/QE7P5.
